# HiMIC-Monthly: A 1 km high-resolution atmospheric moisture index collection over China, 2003–2020

**DOI:** 10.1038/s41597-024-03230-2

**Published:** 2024-04-24

**Authors:** Hui Zhang, Ming Luo, Wenfeng Zhan, Yongquan Zhao, Yuanjian Yang, Erjia Ge, Guicai Ning, Jing Cong

**Affiliations:** 1 Guangdong Provincial Key Laboratory of Urbanization and Geo-simulation, School of Geography and Planning, Sun Yat-sen University, Guangzhou, 51006 China; 2 Institute of Environment, Energy and Sustainability, The Chinese University of Hong Kong, Shatin, Hong Kong SAR China; 3 Jiangsu Provincial Key Laboratory of Geographic Information Science and Technology, International Institute for Earth System Science, Nanjing University, Nanjing, 210023 China; 4 Key Laboratory of Watershed Geographic Sciences, Nanjing Institute of Geography and Limnology, Chinese Academy of Sciences, Nanjing, 210008 China; 5 School of Atmospheric Physics, Nanjing University of Information Science & Technology, Nanjing, 210044 China; 6 Dalla Lana School of Public Health, University of Toronto, Toronto, Ontario M5T 3M7 Canada; 7 Tianjin Municipal Meteorological Observatory, Tianjin, 300074 China

**Keywords:** Environmental sciences, Climate sciences

## Abstract

Near-surface atmospheric moisture is a key environmental and hydro-climatic variable that has significant implications for the natural and human systems. However, high-resolution moisture data are severely lacking for fine-scale studies. Here, we develop the first 1 km high spatial resolution dataset of monthly moisture index collection in China (HiMIC-Monthly) over a long period of 2003~2020. HiMIC-Monthly is generated by the light gradient boosting machine algorithm (LightGBM) based on observations at 2,419 weather stations and multiple covariates, including land surface temperature, vapor pressure, land cover, impervious surface proportion, population density, and topography. This collection includes six commonly used moisture indices, enabling fine-scale assessment of moisture conditions from different perspectives. Results show that the HiMIC-Monthly dataset has a good performance, with R^2^ values for all six moisture indices exceeding 0.96 and root mean square error and mean absolute error values within a reasonable range. The dataset exhibits high consistency with *in situ* observations over various spatial and temporal regimes, demonstrating broad applicability and strong reliability.

## Background & Summary

Atmospheric water vapor is a fundamental component of the Earth’s climate system^1^ and a primary constituent of greenhouse gases^2^, exerting important impacts on climate and environment changes at global and regional scales^3–5^. Especially, near-surface atmospheric moisture plays a vital role in regulating the exchange of energy and moisture between the Earth’s surface and the atmosphere^6,7^, with far-reaching impacts on both human society and ecosystems^8^. Near-surface atmospheric moisture affects hydrological cycles, precipitation patterns, and tropical cyclones^9^, as well as snow melting^10^ and plant growth^11^. Changes in near-surface atmospheric moisture levels have significant implications for the human living environment and public health^12^. For example, under hot weather conditions, increased humidity levels can impede the body’s ability to dissipate heat through sweating, exacerbating the risk of heat exhaustion and its related illnesses^13–15^. In addition, high humidity and temperature can exacerbate the negative effects of air pollution^16^. Changes in humidity patterns may also favor the spread of diseases such as influenza^17^, malaria, and dengue fever^18^. Therefore, accurate measurement of near-surface atmospheric moisture is an important basis for understanding climate change, natural ecosystems, and human society.

Near-surface atmospheric moisture varies significantly across both time and space because of the spatiotemporal variations with related factors including land surface properties, topography, and atmospheric conditions. Atmospheric moisture can be directly measured *in situ* and obtained from climate modeling, but it cannot be easily retrieved from remote sensing technology which typically provides information on column moisture concentration. Several products of near-surface atmospheric moisture indicators at various spatial and temporal resolutions have been developed. These products covering the globe or China can be categorized into four groups (Table 1): climate reanalysis (e.g., ERA5^19^, ERA5-Land^20^, MERRA-2^21^, and NCEP/NCAR^22^), interpolation (e.g., HadCRUH^23^ and HadISDH^24^), data assimilation (e.g., GLDAS^25^), and data fusion (e.g., CMFD^26^). These datasets offer a high temporal resolution (e.g., sub-daily), but their spatial resolution is coarse (i.e., 0.1° ~ 5°, see Table 1). The lack of a high spatial resolution dataset remains a barrier to fine-scale research. There is an urgent need for more accurate and fine-scale moisture datasets.

**Table 1:** A summary of previously developed dataset associated with near-surface atmospheric moisture.

Category	Dataset	Horizontal coverage	Spatial resolution	temporal coverage	Temporal resolution	Variables	Source
Interpolation	HadCRUH	Global	5° × 5°	Monthly	1973 ~ 2003	SH, RH	Willett *et al*.^23^
Interpolation	HadISDH	Global	5° × 5°	Monthly	1973 ~ 2021	SH, RH, DPT, AVP	Willett *et al*.^24^
Climate Reanalysis	ERA5	Global	0.25° × 0.25°	Hourly	1940 ~ present	DPT	Hersbach^19^
Climate Reanalysis	ERA5-Land	Global	0.1° × 0.1°	Hourly	1950 ~ present	DPT	Muñoz Sabater^20^
Climate Reanalysis	MERRA-2	Global	0.6° × 0.25°	Hourly	2006 ~ 2016	AVP	Suarez *et al*.^21^
Climate Reanalysis	NCEP/NCAR	Global	2.5° × 2.5°	6 Hourly	1948 ~ present	RH, SH	Kalnay *et al*.^22^
Assimilation	GLDAS	Global	0.25° × 0.25°	3 Hourly	2000 ~ present	AVP	Rodell *et al*.^25^
Fusion	CMFD	China	0.1° × 0.1°	3 Hourly	1979 ~ 2018	SH	He *et al*.^26^

Various indicators have been proposed to measure the level of atmospheric moisture. Commonly used indicators can be classified into relative and absolute groups. The former group includes relative humidity (RH) and vapor pressure deficit (VPD), and the latter contains dew point temperature (DPT), actual vapor pressure (AVP), mixing ratio (MR), and specific humidity (SH). These indicators reflect the different perspectives of atmospheric moisture and can be used in various fields. For example, RH has been commonly used for human and animal health^27^ and air quality monitoring^28^. VPD is a critical variable in studies of vegetation growth^11^, wildfires^29^, and drought and atmospheric aridity^30^. SH is commonly employed to calculate the total precipitable water in air column and to quantify the transport of water vapor^8^. However, no universal indicator can fully capture the complexity of near-surface atmospheric moisture, and a high spatial resolution dataset with multiple moisture indices is thus urgently needed.

An accurate and fine-scale atmospheric moisture dataset is a basic requirement to support urban climate, regional environment, and human health studies. To date, however, there is no high spatial resolution (e.g., 1 km) dataset with multiple moisture indicators. To fill this gap, the current study aims to construct a Chinese atmospheric moisture dataset with multiple indicators at a high spatial resolution (1 km × 1 km), employing a machine learning algorithm based on multi-source datasets. The main research objectives of this study are: (1) to construct high spatial resolution atmospheric moisture prediction models using data from multiple sources; (2) to evaluate the accuracy and applicability of atmospheric moisture models at different spatiotemporal regimes; (3) to investigate the spatial and temporal changes of atmospheric moisture in China.

## Methods

### Station observation data

**Fig. 1:**
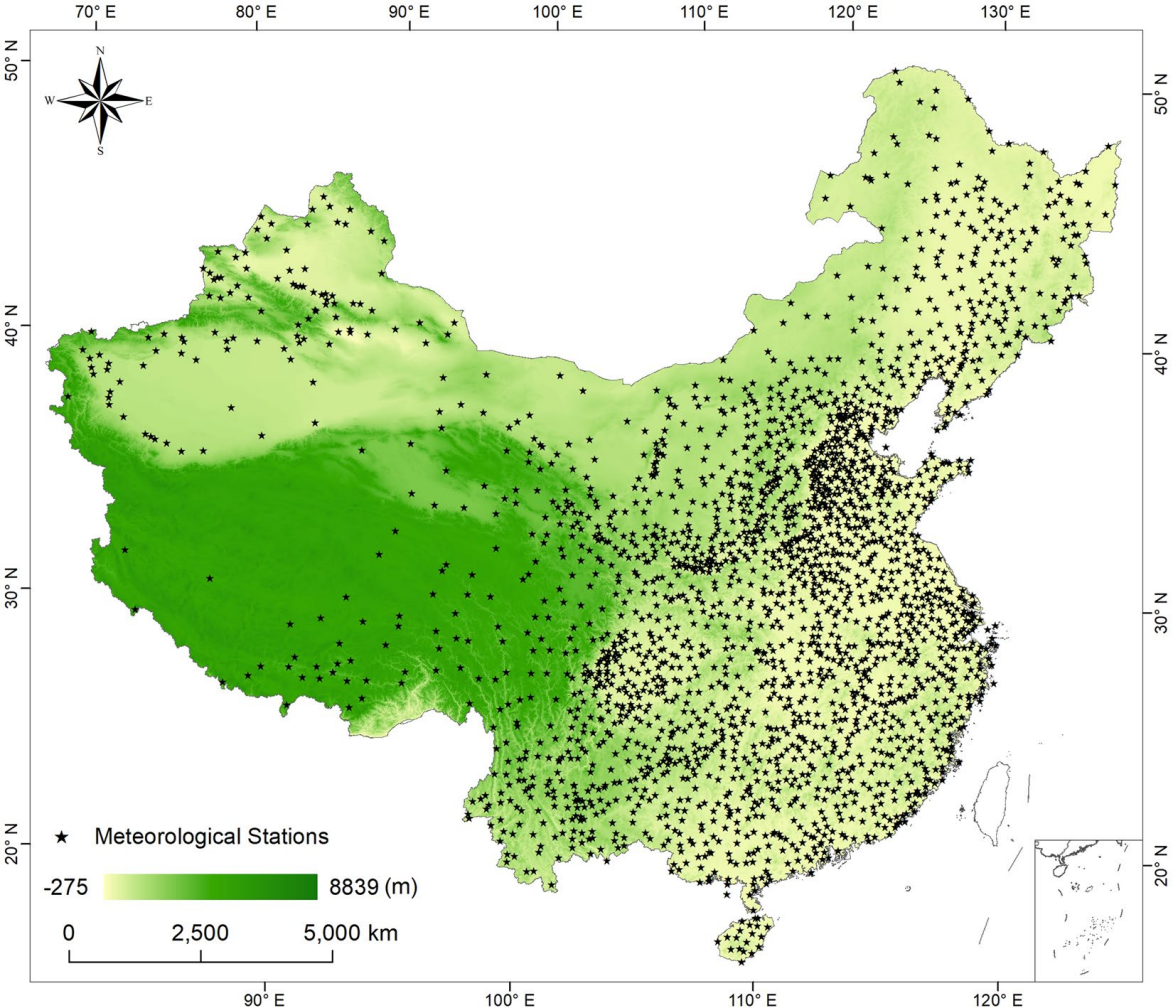
Spatial distribution of weather stations in the mainland of China, with color shading indicating the elevation in meters.

*In situ* observations at 2,419 meteorological stations across the mainland of China were collected from the China Meteorological Data Service Centre (http://data.cma.cn/) of the China Meteorological Administration (CMA) from January 2003 to December 2020. The spatial distribution of these meteorological stations is shown in Fig. 1, and detailed information on stations can be found at https://zenodo.org/records/10612781. The recorded variables include daily mean air temperature (SAT), RH, and surface pressure (PRS). All records collected from these stations underwent a rigorous quality control and evaluation process by CMA^31^. In accordance with the terms of use specified by CMA, the station observation data utilized in this study are not permitted for redistribution. Readers interested in directly accessing the data are encouraged to refer to the official channels provided by CMA for data acquisition and usage permissions.

### Covariates

The spatiotemporal variations of near-surface atmospheric moisture are closely related to land surface properties, topography, atmospheric conditions, and human activities. In this study, land surface temperature (LST), vapor pressure, land cover, elevation, slope, the proportion of impervious surface, population density, the month of the year, and year are selected as the covariates to predict six commonly used moisture indicators (Table 2).

**Table 2:** Gridded datasets and covariates used to predict near-surface atmospheric moisture indices.

Category	Dataset	Spatial Resolution	Temporal Resolution	Variables	Data Source
Land surface temperature	A global seamless 1 km resolution daily land surface temperature dataset (2003–2020)	1 km	Daily	Land surface temperature	Zhang *et al*.^35^
Vapor Pressure	TerraClimate	4 km	Monthly	Resampled vapor pressure in 1 km	Abatzoglou *et al*.^36^
Land cover	MCD12Q1.006	500 m	Annual	Land cover classes in 1 km grids	Sulla-Menashe and Friedl^37^
Impervious surface	GAIA	30 m	Annual	Proportion of impervious surface in 1 km grids	Gong *et al*.^43^
Population density	WorldPop	1 km	Annual	Population density	Gaughan *et al*.^44^
Topography	MERIT	90 m	/	Aggregated elevation and slope in 1 km grids	Yamazaki *et al*.^45^
Temporal variation	/	/	/	Month of the year, Year	/

LST plays a crucial role in modulating near-surface atmospheric moisture through several mechanisms^32–34^. As LST increases, the rate of evaporation of water from the land surface increases, leading to a subsequent increase in near-surface atmospheric moisture content. Warmer LST may increase the height of the atmospheric boundary layer, resulting in more mixing of air and moisture from different levels of the atmosphere, thus increasing near-surface atmospheric moisture. Also, the LST changes can impact atmospheric circulation patterns, which can subsequently affect the transport and distribution of moisture in the atmosphere. The daily LST dataset at 1 km × 1 km spatial resolution from 2003 to 2020 is obtained from Zhang *et al*.^35^. This dataset was derived from the Moderate Resolution Imaging Spectroradiometer (MODIS) LST product and included both daytime and nighttime estimates. This dataset was generated using a suite of algorithms that incorporate atmospheric correction, cloud and snow masking, and spatiotemporal gap-filling algorithm, and shows good agreement with observations. The LST value of each pixel comprises two components: the overall trend and the daily fluctuations^35^. This gap-filling method involves initially using a smoothing spline function to fit the overall trend of each pixel for each day. Subsequently, the inverse distance weighting interpolation method is applied to interpolate spatiotemporal residuals. The final gap-filled LST values of the pixel are obtained by summing the corresponding trend and residuals^35^.

The vapor pressure data are obtained from the TerraClimate dataset developed by Abatzoglou *et al*.^36^. The temporal and spatial resolutions of the TerraClimate dataset are 1 month and 1/24° (~4 km), respectively. This dataset was generated by integrating multiple climate datasets and utilizing climatically aided interpolation techniques, resulting in a significant improvement in accuracy compared with the datasets with coarser spatial resolutions. In our study, monthly vapor pressure is interpolated to 1 km × 1 km spatial resolution using the bilinear method.

Global land cover types at a spatial resolution of 500 m are fetched from the MCD12Q1.006 dataset^37^. This dataset was produced by combining data from the MODIS sensors aboard the Terra and Aqua satellites with other ancillary datasets and utilizing a supervised classification algorithm, and it has been widely used in ecological and environmental research^38,39^, disaster management^40^, and climate modeling^41,42^. The global artificial impervious area (GAIA) dataset at a high spatial resolution of 30 m was produced by Gong *et al*.^43^, and the population density dataset was collected from the WorldPop project^44^.

Furthermore, the spatial distribution of near-surface atmospheric moisture is closely related to topography, particularly elevation and slope. Therefore, the Multi-Error-Removed Improved-Terrain (MERIT) dataset with a spatial resolution of 3 arc seconds (~ 90 m) obtained from Yamazaki *et al*.^45^ is used in our study. As near-surface atmospheric moisture exhibits different changes across years and months, both year and the month of the year are also considered as covariates. Considering that incorporating wind speed may lower the model performance (Supplementary Table 1), we do not include wind speed as a covariate. A detailed summary of the covariates and datasets used in the study is provided in Table 2.

### Methodology

**Fig. 2:**
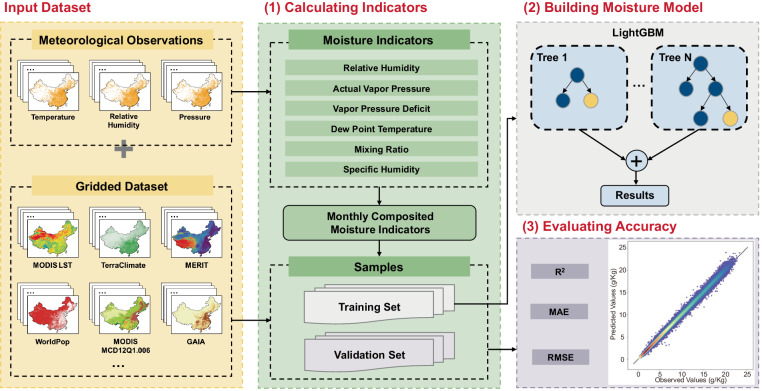
The framework for developing the HiMIC-Monthly dataset.

The workflow developed for constructing the atmospheric moisture dataset by a machine learning algorithm based on multi-source datasets is depicted in Fig. 2. The approach consists of three major parts. First, daily atmospheric moisture indices are computed using observation records, and are then aggregated on a monthly basis. Second, the construction and optimization of the atmospheric moisture prediction model are carried out using the Light Gradient Boosting Model (LightGBM) algorithm. Third, the accuracy of prediction is evaluated using three commonly used metrics.

### Calculation of atmospheric moisture indices

Six commonly used near-surface atmospheric moisture indices including RH, AVP, VPD, DPT, MR, and SH are predicted in our study, and their calculations are summarized in Table 3. All indices are initially computed on a daily basis, followed by the derivation of monthly means by averaging the corresponding daily values within their respective months. It is emphasized that the calculation of RH and VPD involves saturation vapor pressure (SVP, unit: hPa; Murray^46^):

1 $$SVP=6.112\times ex{p}^{\frac{17.67\times SAT}{SAT+243.5}}$$

where SAT is the surface air temperature at 2 m above the ground (unit: °C).

**Table 3:** Calculation of near-surface atmospheric moisture indices. SVP: saturation vapor pressure (unit: hPa), PRS: surface pressure (unit: hPa).

Moisture Indices	Abbreviation	Formula	Unit	Citation
Relative Humidity	RH	$RH=100\times AVP/SVP$	%	Murray^46^
Actual Vapor Pressure	AVP	$AVP=RH\times SVP/100$	hPa	Murray^46^
Vapor Pressure Deficit	VPD	$VPD=SVP-AVP$	hPa	Buck^69^
Dew Point Temperature	DPT	$DPT=\mathrm{log}(AVP/6.112)\times 243.5/(17.67-\mathrm{log}(AVP/6.112))$	°C	Bolton^70^
Mixing Ratio	MR	$MR=\frac{0.62197\times AVP}{PRS-AVP}\times 1000$	g/kg	Salby^71^
Specific Humidity	SH	$SH=\frac{MR}{1+MR}\times 1000$	g/kg	Salby^71^

### Prediction of atmospheric moisture indices

LightGBM algorithm developed by Ke *et al*.^47^ is employed in our study to predict atmospheric moisture indices. LightGBM is a popular machine learning algorithm that has gained much attention due to its high efficiency and accuracy. It is a gradient boosting framework using a tree-based learning algorithm, which is designed to be distributed and efficient. Compared with other algorithms, such as eXtreme Gradient Boosting (XGBoost) and Categorical Boosting (CatBoost), LightGBM has faster speed and higher rates of accuracy^48^ by introducing the leaf-wise growth strategy. This strategy grows the tree by selecting the leaf with the maximum delta loss to split, which leads to a higher accuracy at the cost of a slightly longer training time. It also uses the Gradient-based One-Side Sampling (GOSS) to select important categorical features and reduce the dimensionality of the problem. Its high accuracy and stability have been substantiated in building prediction models for both classification and regression tasks of geophysical variables^49–51^.

LightGBM algorithm is implemented using the Python library LightGBM (https://lightgbm.readthedocs.io/en/latest/Python-Intro.html). In this study, the observations of monthly moisture indices are divided into a training set (80%) and a validation set (20%) in a random manner, serving the purposes of model training and assessment, respectively. The optimization of training model performance critically relies on the selection of appropriate hyperparameters. Hence, a grid search method coupled with 5-fold cross-validation is employed to fine-tune the hyperparameters, aiming to identify the best parameter configuration based on the evaluation metric of Root Mean Square Error (RMSE).

### Assessment of accuracy

The performance of the dataset produced in this study is verified using three metrics, i.e., coefficient of determination (R^2^), RMSE, and mean absolute error (MAE). These metrics have been extensively employed to assess the accuracy and precision of regression models^35,52,53^, and provide a comprehensive evaluation of the dataset. The R^2^ metric is employed to evaluate the goodness-of-fit of the regression model, ranging from 0 to 1 (perfect fit). The RMSE and MAE metrics, on the other hand, are used to quantify the bias between the observed values and the corresponding predicted values. The computation of these three metrics is based on the following equations:

2 $${R}^{2}=1-\frac{{\sum }_{i=1}^{N}{({y}_{i}-{\widehat{y}}_{i})}^{2}}{{\sum }_{i=1}^{N}{({y}_{i}-\bar{\mathrm{y}})}^{2}}$$

3 $$RMSE=\sqrt{\frac{1}{N}\times \mathop{\sum }\limits_{i=1}^{N}{\left({y}_{i}-{\widehat{y}}_{i}\right)}^{2}}$$

4 $$MAE=\frac{1}{N}\times \mathop{\sum }\limits_{i=1}^{N}\left|{y}_{i}-{\widehat{y}}_{i}\right|$$

where *y*_*i*_ is the observed value of moisture indices, ${\widehat{y}}_{i}$ is the predicted value of moisture indices, $\bar{y}$ is the mean of the observed value of moisture indices calculated from meteorological stations, and *N* is the number of samples.

## Data Records

The HiMIC-Monthly dataset, spanning from January 2003 to December 2020, is freely available from Zenodo at https://zenodo.org/record/8070140^54^, and the National Tibetan Plateau Data Center of China at https://data.tpdc.ac.cn/zh-hans/data/6854ebb3-8a60-454a-8d43-4e6a8c0ebd5d. The dataset is stored in NetCDF and GeoTIFF file formats. It includes six moisture indices, namely RH (0.01%), AVP (0.01 hPa), VPD (0.01 hPa), DPT (0.01 °C), MR (0.01 g/kg), and SH (0.01 g/kg). It covers the mainland of China with a high spatial resolution of 1 km × 1 km and a coordinate system of Albers equal-area conic projection. This dataset is organized and compressed on a yearly basis, with each zip package or stack containing 12 monthly images. All moisture values are multiplied by 100 and stored as an integer (Int16) to save storage space. When in use, these values need to be divided by 100 to obtain the corresponding units in %, hPa, hPa, °C, g/kg, and g/kg for RH, AVP, VPD, DPT, MR, and SH, respectively. Additional information on the dataset can be found in “README.pdf”.

## Technical Validation

### Overall accuracy assessment

**Fig. 3:**
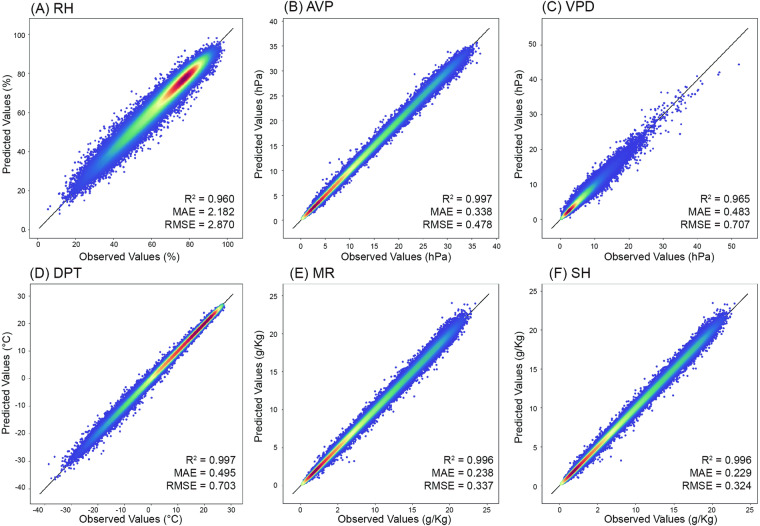
Performance of the LightGBM models for six moisture indices over the mainland of China during 2003~2020: (**A**) RH, (**B**) AVP, (**C**) VPD, (**D**) VPD, (**E**) MR, and (**F**) SH. The color represents the density of data points, in which the red (blue) dots represent the highest (lowest) density. The black line represents the 1:1 line.

Our predicted moisture indices have high accuracy with R^2^ values above 0.96 (Table 4). Specifically, the R^2^ values of AVP, DPT, MR, and SH are higher than 0.99. The scatterplots of the observed and predicted values for six moisture indices are presented in Fig. 3. The predicted moisture indices by the LightGBM model are in good agreement with *in situ* observational data, as the predicted and observed values of moisture indices concentrate along the 1:1 line. Moreover, the MAE and RMSE values of the six moisture indices are within a reasonable range. The MAE and RMSE values of RH are lower than 2.18% and 2.87%, respectively. AVP has MAE and RMSE values of 0.34 hPa and 0.48 hPa, respectively. VPD receives MAE and RMSE values of 0.48 hPa and 0.71 hPa, respectively. The MAE and RMSE values of DPT are 0.49 °C and 0.70 °C, respectively. The MAE and RMSE values of MR are 0.24 g/kg and 0.34 g/kg, respectively. The MAE and RMSE of SH are 0.23 g/kg and 0.32 g/kg, respectively. These results suggest that the predicted six moisture indices are of good quality and are suitable for fine-scale studies.

**Table 4:** Overall accuracies of the six moisture indices from 2003 to 2020.

Moisture Indices	R^2^	MAE	RMSE
RH (%)	0.960	2.182	2.870
AVP (hPa)	0.997	0.338	0.478
VPD (hPa)	0.965	0.483	0.707
DPT (°C)	0.997	0.495	0.703
MR (g/kg)	0.996	0.238	0.337
SH (g/kg)	0.996	0.229	0.324

Furthermore, the prediction accuracy of the LightGBM model is compared with three commonly used machine learning algorithms, including XGBoost^55^, CatBoost^56^, and Random Forest^57,58^ (Supplementary Table 2), and we find that the LightGBM exhibits the best performance in terms of the highest R^2^ and the lowest MAE and RMSE values. We further assess the ability of LightGBM by conducting an independent round of validation. We leave out ~5‰ (five per thousand) of randomly selected stations and estimate the moisture level of these left-out stations by using the observations at other stations. This process is repeated 200 times for each moisture indicator, and thus the metrics of R^2^, MAE, and RMSE for all stations can be obtained. The results are shown in Supplementary Table 3, which indicates that the R^2^ values of six predicted indices are higher than 0.86. The MAE and RMSE values of RH are below 4.031% and 5.335%, respectively, while those of AVP are below 0.664 hPa and 0.944 hPa, respectively. The MAE and RMSE values of VPD are lower than 0.904 hPa and 1.332 hPa, respectively, while those of DPT are lower than 0.943 °C and 1.357 °C, respectively. MR demonstrates MAE and RMSE values below 0.461 g/Kg and 0.658 g/Kg, respectively, and SH exhibits MAE and RMSE values below 0.448 g/Kg and 0.642 g/Kg, respectively. These results demonstrate the superior ability of the LightGBM model.

### Covariate importance

**Fig. 4:**
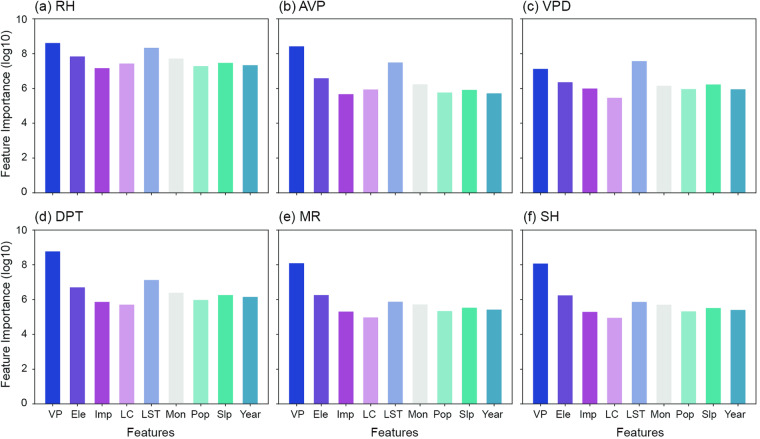
The importance of nine covariates in predicting six moisture indices: (**a**) RH, (**b**) AVP, (**c**) VPD, (**d**) DPT, (**e**) MR, and (**f**) SH. VP, Ele, Imp, LC, LST, Mon, Pop, Slp, and Year represent vapor pressure, elevation, impervious surface, land cover, land surface temperature, month of the year, population density, slope, and year, respectively. The feature importance values are presented in a logarithmic scale, i.e., log(10).

To determine the most influential covariates in predicting the six moisture indices, we conduct a comparative analysis of the feature importance across each model. Vapor pressure acts as the most significant variable in nearly all models (except for VPD, Fig. 4 & Supplementary Fig. 1). LST plays a significant role as a secondary variable in models predicting RH, AVP, and DPT, while elevation emerges as a secondary variable for MR and SH. For predicting VPD, the most crucial factor is identified as LST, followed by vapor pressure, and elevation.

### Spatial distribution of accuracies

**Fig. 5:**
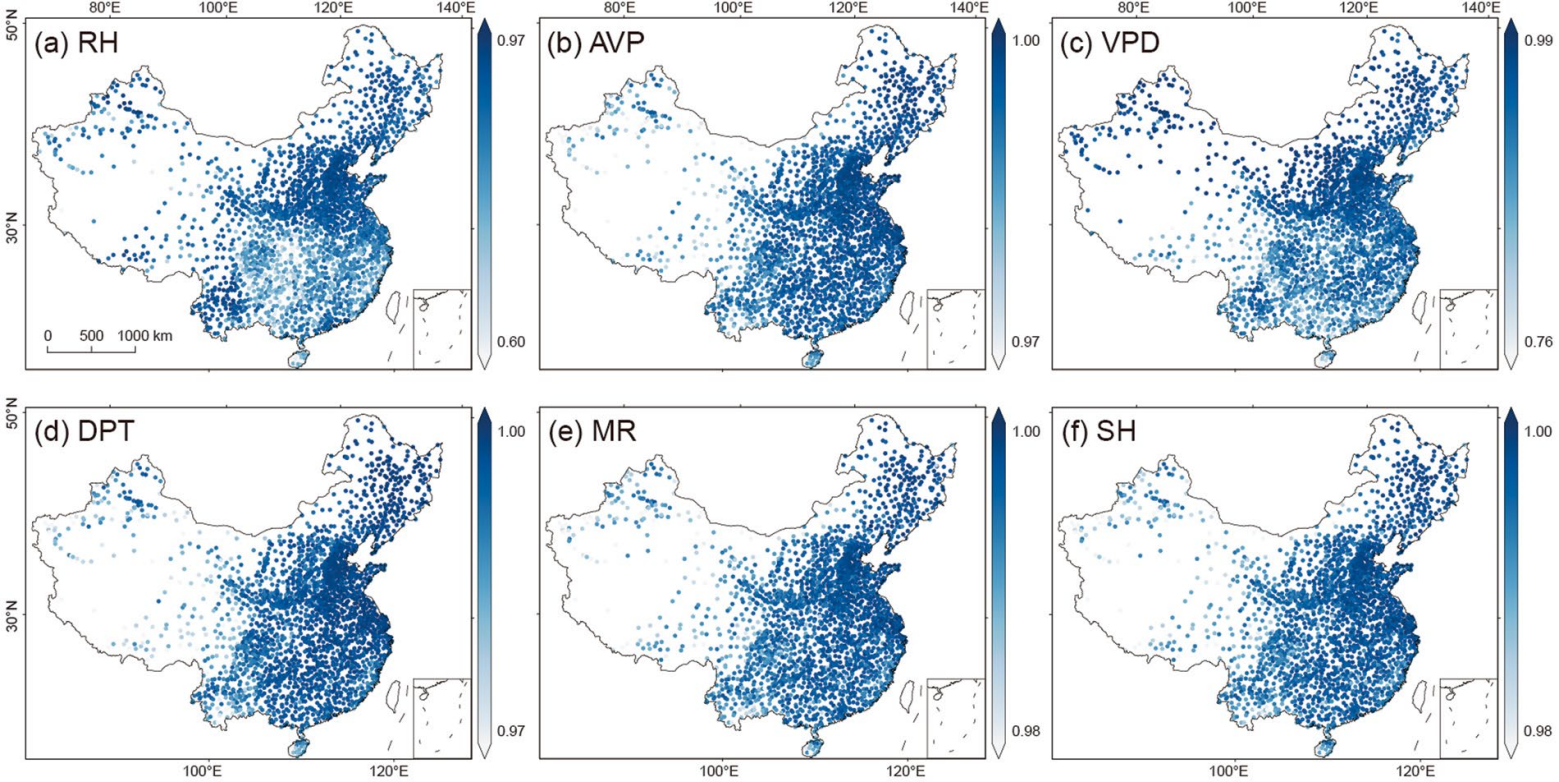
Spatial distribution of R^2^ of the predicted six moisture indices at individual stations across the mainland of China during 2003~2020: (**a**) RH, (**b**) AVP, (**c**) VPD, (**d**) VPD, (**e**) MR, and (**f**) SH.

**Fig. 6:**
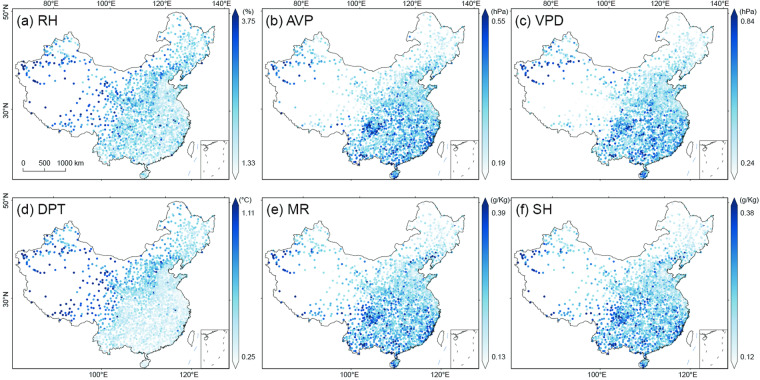
As Fig. 5 but for MAE.

**Fig. 7:**
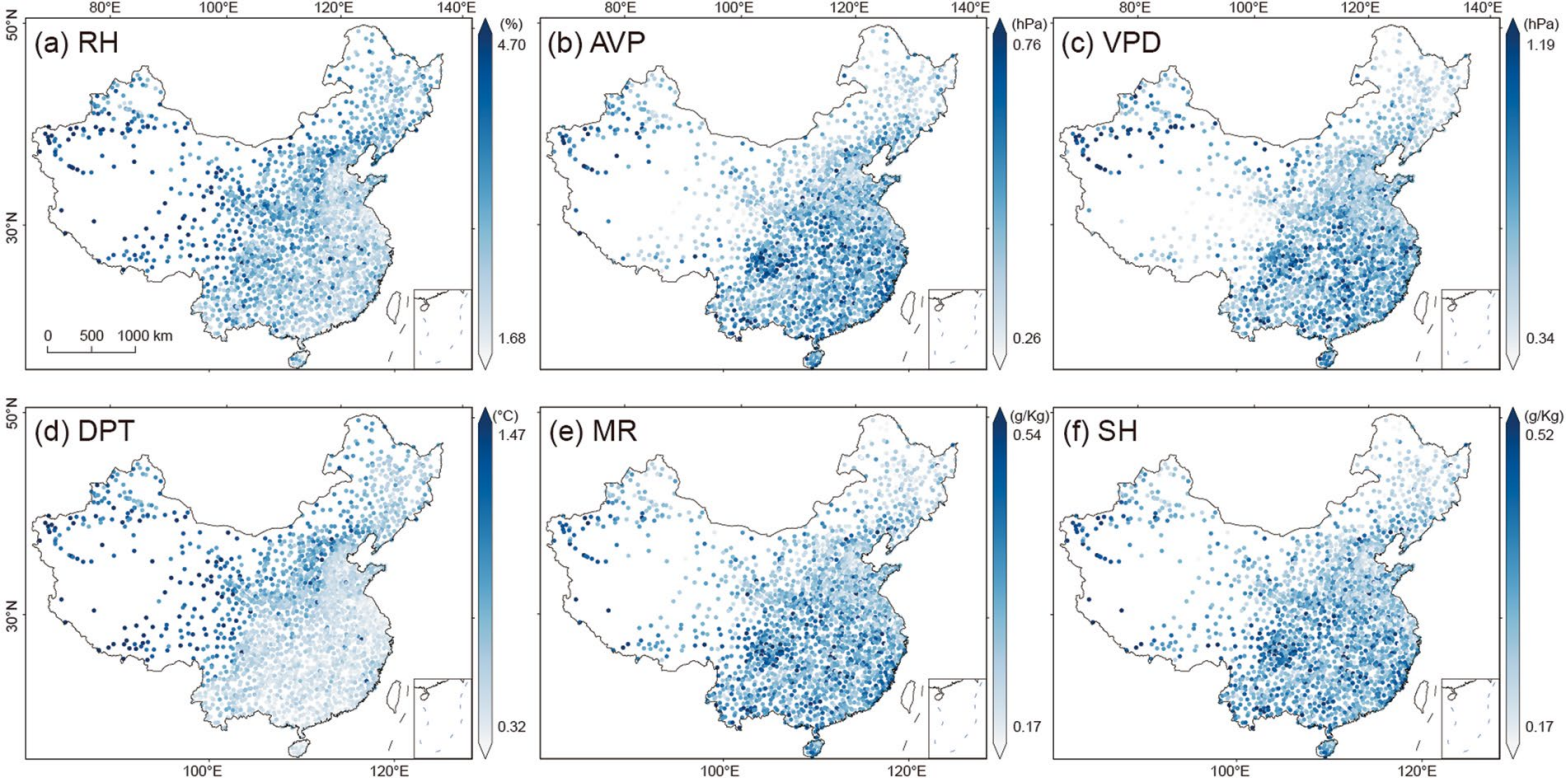
As Fig. 5 but for RMSE.

To gain a more comprehensive understanding of the spatial distribution of the model performance, we map the spatial distributions of R^2^, MAE, and RMSE at individual stations across the mainland of China in Fig. 5–7, respectively. The results exhibit a high consistency with the observations at nearly all individual stations for six moisture indices. The spatial patterns of R^2^ values of AVP, DPT, MR, and SH are similar, with higher R^2^ values (i.e., >0.99) distributed in eastern and northern China and relatively lower in southwestern China. Of RH and VPD, the higher R^2^ values (i.e., >0.95) are mainly located in northern China (e.g., the North China Plain) and Yunnan, while the lower R^2^ values are distributed in southern China.

The MAE and RMSE values are small at nearly all stations (Figs. 6, 7). RH and DPT exhibit a similar spatial distribution of MAE, and higher values are distributed in the west of the Hu Huanyong Line and lower values in the east (Fig. 6). The MAE values of AVP, VPD, MR, and SH show a spatial pattern of higher values in northern China and lower in southeastern China (Fig. 6). Figure 7 displays the spatial distribution of RMSE values of the six moisture indices, and these distribution patterns are consistent with those of MAE.

### Accuracy assessment in individual years and months

We also evaluate the model performance at different time regimes (i.e., year and month). The MAE and RMSE values at the annual scale for six moisture indices are presented in Supplementary Tables 4, 5. The MAE and RMSE exhibit minor variations from year to year during 2003~2020, with relatively lower values appearing in 2016~2017 (Supplementary Figs. 2, 3). The MAE (RMSE) values of RH are within the range of 1.88% ~ 2.41% (2.46% ~ 3.14%). The MAE (RMSE) values of AVP range from 0.27 to 0.39 hPa (0.38 ~ 0.56 hPa), while those of VPD are within the range of 0.44 ~ 0.54 hPa (0.64 ~ 0.81 hPa). The MAE (RMSE) values of DPT are within the range of 0.38 ~ 0.55 °C (0.52 ~ 0.77 °C), and those of MR and SH are within the range of 0.19 ~ 0.28 g/kg and 0.19 ~ 0.27 g/kg. Furthermore, we evaluate the monthly accuracy of six moisture indices (Supplementary Tables 6, 7). The MAE and RMSE values of AVP, VPD, MR, and SH reach their maximum values in summer and minimum in winter, whereas those of RH and DPT exhibit their maximum values in winter and minimum in summer (Supplementary Figs. 4, 5). The variations in MAE and RMSE at annual or monthly scales are within reasonable ranges, indicating that the LightGBM model has good performance and our predicted HiMIC dataset has good reliability at various time scales.

### Accuracy assessment in different climate zones

We further evaluate the accuracies of six predicted moisture indices in nine different climate zones of China (Supplementary Fig. 6 & Tables 8–10). In nearly all zones, all moisture indices exhibit high R^2^ values (i.e., ≥ 0.84, Supplementary Table 10). Especially, the highest R^2^ value (0.955) of RH is seen in the warm temperate zone, and the lowest (0.845) is in the mid-tropical zone. The highest R^2^ value of VPD is observed in the mid-temperate zone, and the lowest (0.840) appears in the mid-tropical zone. The R^2^ values of AVP, DPT, MR, and SH in all climate zones are all higher than 0.984. The MAE values of six predicted moisture indicators exhibit a similar pattern to the RMSE values (Supplementary Tables 8, 9). The lowest MAE (RMSE) values of AVP, VPD, MR, and SH are seen in the cold temperature zone, while lower values are mainly distributed in the mid-tropical zone (Supplementary Tables 8, 9). The highest MAE (RMSE) values of RH and DPT are found in the plateau zone, while the lowest of RH is in the cold temperature zone and that of DPT is in the mid-tropical zone (Supplementary Tables 8, 9). It should also be noted that for sparsely monitored areas further evaluation is still needed, such as including more on-site measurements or incorporating observations from various sources that provide moisture observations (e.g., flux towers stations).

### Accuracy assessment in major urban agglomerations

As the majority of the Chinese population resides in urban areas, it is crucial to evaluate the accuracy of the moisture dataset in urban agglomerations (UAs). Such an evaluation is important to understanding the impact of the ambient environment on urban residents. In this study, we further assess the accuracies of our HiMIC-Monthly dataset in the 20 major UAs of China (Wang *et al*.^59^, Supplementary Tables 11–13). For all six moisture indices, nearly all UAs exhibit high values of R^2^, with an average value of 0.97 (Supplementary Table 11). The highest MAE value of RH is located in the Lanzhou-Xining UA, while that of AVP is located on the West Coast of Taiwan Strait UA (Supplementary Table 12). The highest MAE value of VPD is distributed in the Beibu Gulf UA, that of DPT is in the North Tianshan Mountain UA, and that of MR and SH is in the Chendu-Chongqing UA. The highest RMSE value of RH (3.34%) is observed in the Lanzhou-Xining UA (Supplementary Table 13), while that of AVP (0.59 hPa) and VPD (0.837 hPa) is observed in the Chengdu-Chongqing UA. The highest value of DPT (0.86 °C) is shown in the North Tianshan Mountain UA, while that of MR (0.41 g/kg) and SH (0.39 g/kg) is shown in the West Coast of Taiwan Strait UA. These results are in reasonable ranges, suggesting that our predicted HiMIC-Monthly dataset presents a good consistency with observations at the urban scale, providing a scientific basis for urban studies at a fine scale.

### Spatial variations of the predicted moisture indices

**Fig. 8:**
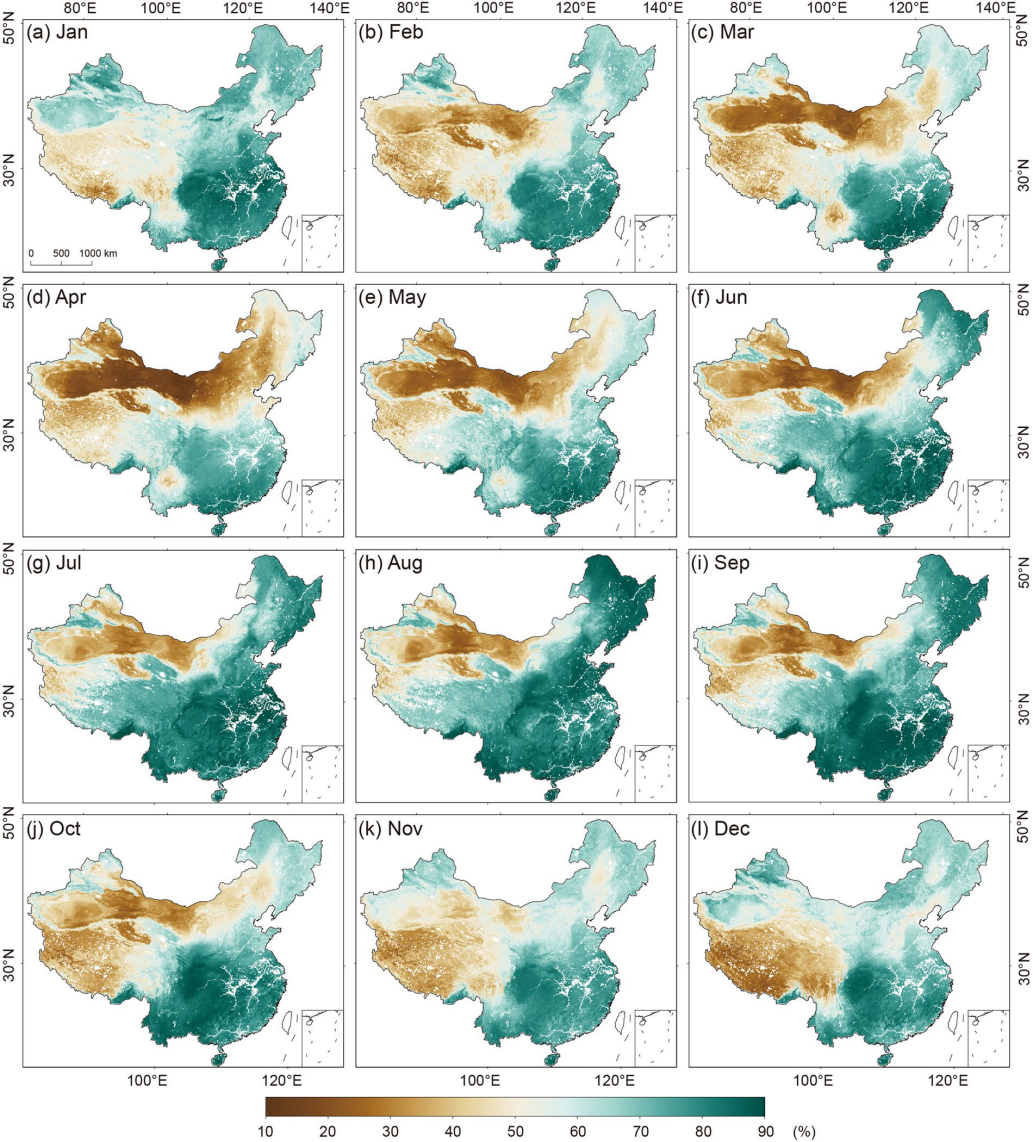
Spatial patterns of the monthly mean RH over the mainland of China in 12 calendar months of 2020.

The above assessments demonstrate that our model exhibits good performance at various spatial (i.e., national and local) and temporal (i.e., yearly and monthly) scales. On this basis, we employ this robust model to generate a high-resolution (1 km × 1 km) and multiple moisture index collection at a monthly scale for China (HiMIC-Monthly) spanning from 2003 to 2020. To illustrate the potential of our dataset, we examine the monthly changes in the spatial distribution patterns of HiMIC-Monthly by taking RH as an example (Fig. 8). RH demonstrates lower values in the northwestern region and higher values in the southeastern region, reflecting the influence of topography, land cover, and climate zones. Specifically, as elevation increases, RH values tend to decrease. From arid to humid regions, RH values tend to increase, with the Taklimakan Desert in arid Northwest China exhibiting the lowest RH values and the Pearl River Delta in humid South China displaying the highest RH values. Moreover, notable temporal variations in the spatial distribution of RH are observed across 12 calendar months. Summer months exhibit higher RH values, while winter months experience lower RH values. These variations in RH throughout different months provide robust evidence for the reliability of our HiMIC-Monthly dataset.

**Fig. 9:**
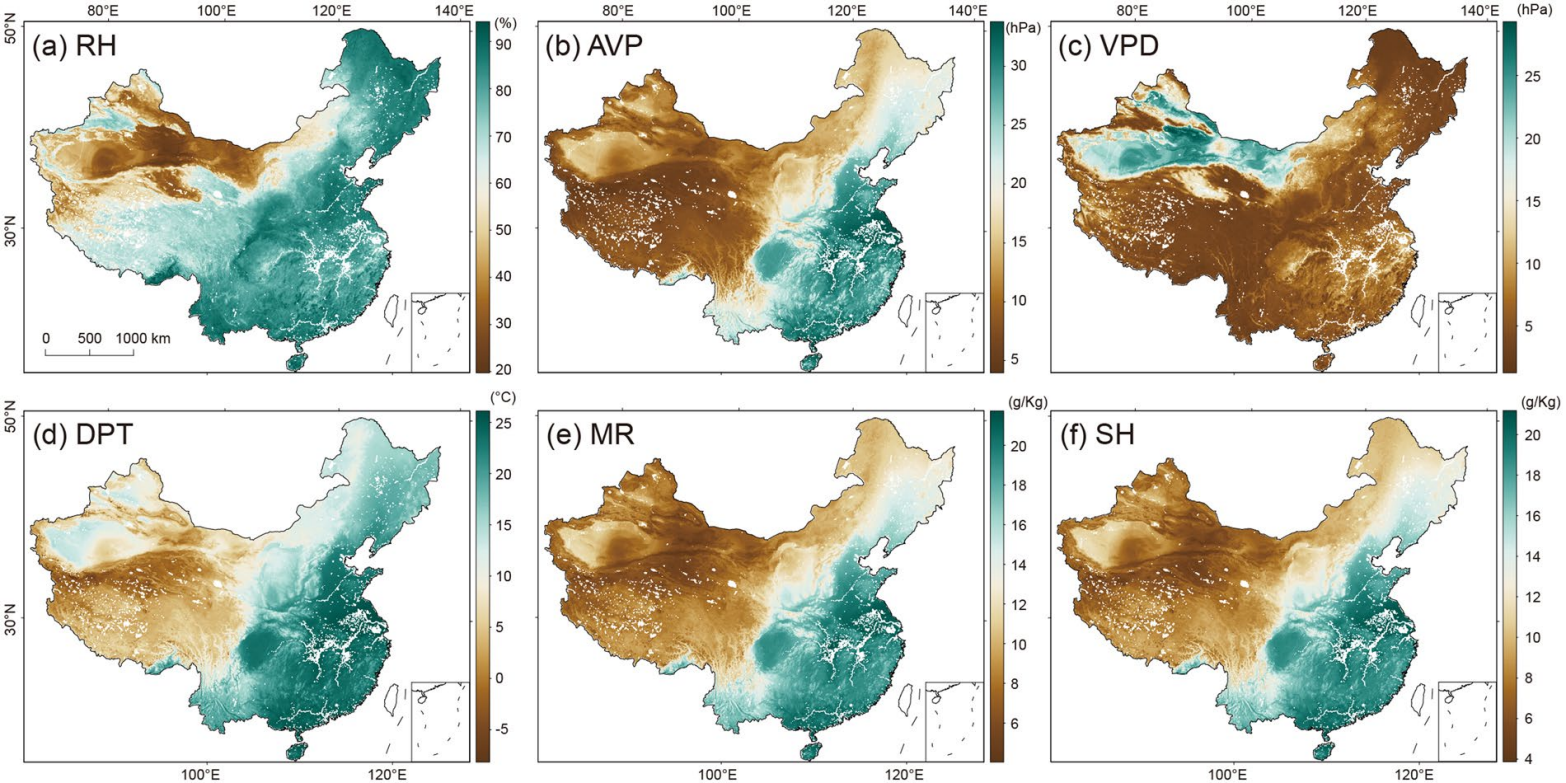
Spatial patterns of the six moisture indices over the mainland of China in August 2020.

Figure 9 displays the spatial distribution of six moisture indices in August of 2020. This particular month was chosen due to the occurrence of persistent heavy rainfall events in China which were listed among the top 10 national natural disasters of 2020 in the country^60^. AVP, DPT, MR, and SH have a similar spatial distribution, with high values mainly distributed in the west of the Hu Huanyong Line, and low values in the east. The high (low) values of RH (VPD) are distributed in southern and eastern China, while low (high) values are located in northwestern China, especially in the Taklimakan desert. These patterns further demonstrate the reliability of our dataset.

### Potentials of the HiMIC-Monthly dataset

The HiMIC-Monthly dataset holds immense potential for various applications. In the field of human society studies, this dataset can be used to study the spatiotemporal changes of fine-resolution human heat stress, on which humidity may induce additional exacerbation^61,62^, the spread and prevalence of various diseases (e.g., respiratory diseases^63^ or vector-borne illnesses^64^) that are under the influences of air moisture conditions. It also enables investigations into the changes in urban dry/wet islands^65^, which may further influence urban air quality at the intra-urban scale and have not been well understood in the literature because of the lack of a fine-scale moisture dataset. Within the field of natural systems, our dataset can play an important role in predicting the growth of plants, whose photosynthesis and evapotranspiration are closely linked to the humidity level in the surrounding atmospheres^66^. It can also be used for estimating crop yield, assessing the suitability of different regions for specific crops, and evaluating the risk of humidity-related crop diseases. In addition, this moisture dataset can provide support for forecasting wildfires^67^ and snowpack ablation^10^.

### Comparison with existing dataset

**Fig. 10:**
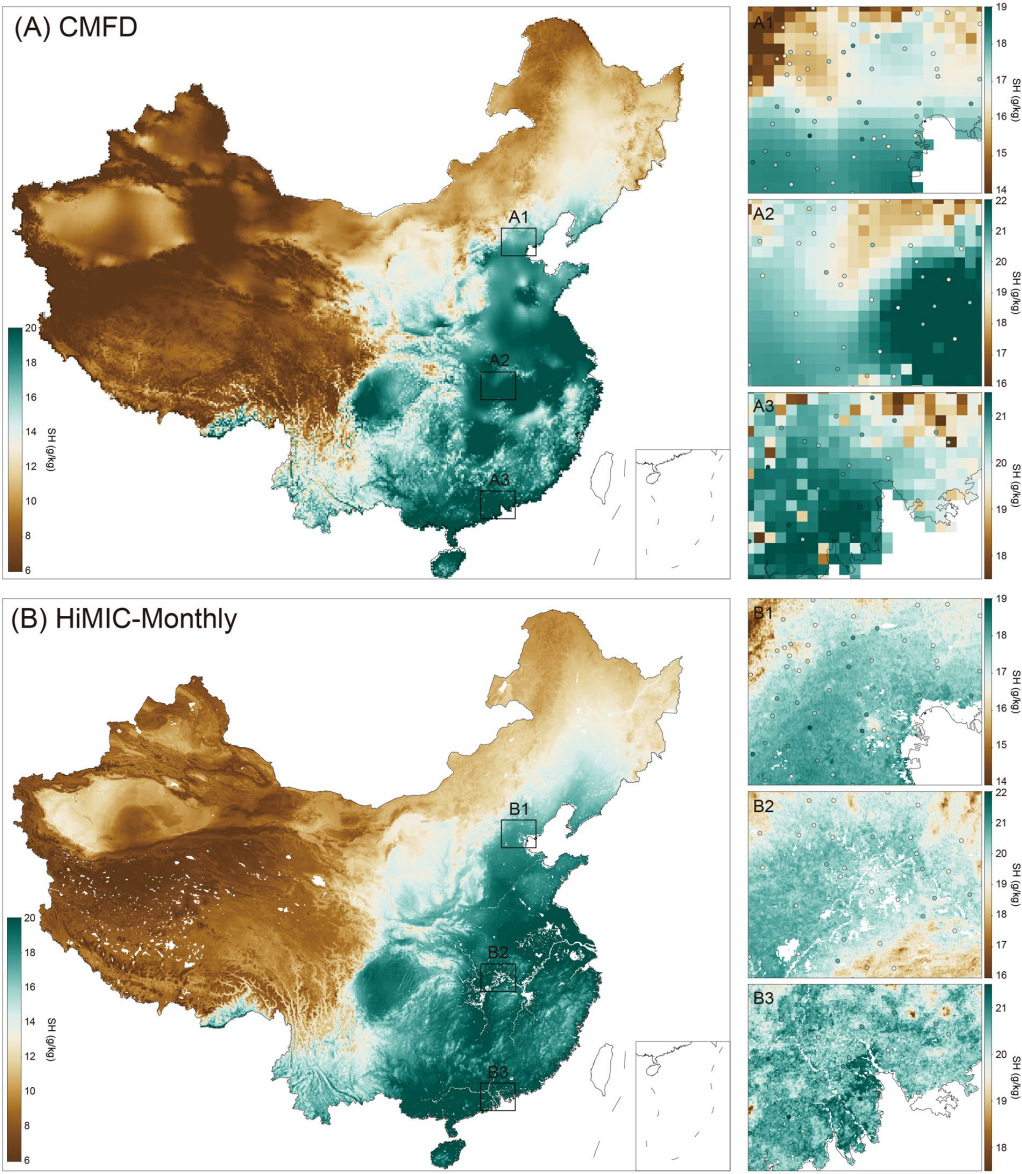
Comparison of the spatial patterns between CMFD and HiMIC-Monthly datasets for SH over the mainland of China and the three largest UAs in August 2018, i.e., A1&B1: Beijing-Tianjin-Hebei, A2&B2: middle Yangtze River Valley, and A3&B3: Pearl River Delta. Colored circles indicate the observed SH (g/kg) values at individual stations.

We further compare the HiMIC-Monthly dataset with an existing product, namely the China Meteorological Forcing Dataset (CMFD, He *et al*.^26^), which has a coarse spatial resolution of 0.1° × 0.1° (Table 1). Comparison is applied to monthly mean SH in August 2018 across China (comparisons in other months are similar and thus not shown), with a particular focus on the three largest UAs: Beijing-Tianjin-Hebei, the middle Yangtze River Valley, and the Pearl River Delta (Fig. 10). Out of the six moisture indices, SH is selected because CMFD does not provide other moisture indicators. The two datasets portray a similar overall spatial pattern of low values in western and northern China and high values in the south (left panel of Fig. 10). Compared with CMFD, however, our HiMIC-Monthly dataset provides much more detailed information on spatial variations (right panel of Fig. 10). While CMFD is able to describe the SH difference between plateaus and plains, it cannot provide detailed spatial information, especially in the intra-city; whilst our HiMIC-Monthly elaborates on the spatial variation of moisture. By comparing the observed values at individual stations, it is also evident that CMFD exhibits numerous overestimations or underestimations of SH values, whereas our HiMIC-Monthly dataset demonstrates a much higher consistency with the observations. These results indicate that our HiMIC-Monthly dataset can effectively and accurately capture the spatial variations in urban areas, thereby providing essential support for fine-scale studies. We further compare the difference in SH between the CMFD and HiMIC-Monthly datasets over the mainland of China from 2003 to 2018 (Supplementary Fig. 7). The SH values of CMFD are lower than those in HiMIC-Monthly in most parts of China, while some higher values in CMFD are observed in small parts of Southwest China, and parts of Southeast and East China.

### Limitations and future works

This study develops a high-resolution and long-term near-surface atmospheric moisture dataset (HiMIC-Monthly), which is useful in studies related to urban climate, environmental science, ecosystems, and public health. Our dataset offers detailed information on multiple moisture indicators at fine spatial scale. In our study, LST and vapor pressure are selected to predict moisture indices. The LST dataset was produced under clear-sky conditions and did not consider the effects of cloud cover. Also, the spatial resolution of the vapor pressure variable is relatively coarse (4 km × 4 km), and is interpolated into 1 km × 1 km. A finer-scale vapor pressure variable can improve the accuracy of predictions.

Our dataset is at a monthly scale, which may not fully meet the need for research on extreme weather events and related environmental issues at a daily scale. Therefore, we are working to develop and release a new collection of high-resolution moisture indices on a daily scale (HiMIC-Daily). In our current study, we provide the first national-level dataset with multiple high-resolution moisture indices for the mainland of China, and this dataset shows desirable accuracies across different climate regimes of China. A global dataset of multiple moisture indices is urgently needed for a wide range of applications in earth system science, land and hydrological models, and the related fields.

### Supplementary information

HiMIC-Monthly: A 1 km high-resolution atmospheric moisture index collection over China, 2003–2020

## Data Availability

Sample codes for developing the HiMIC-Monthly dataset are available from Zenodo^68^ at 10.5281/zenodo.8352539.
