# Supplementary material for: HiMIC-Monthly: A 1 km high-resolution atmospheric moisture index collection over China, 2003–2020
Source: Sci Data. 2024 Apr 24;11:425. doi: 10.1038/s41597-024-03230-2 (PMC11043353; doi:10.1038/s41597-024-03230-2)
Supplement: Supplementary file 1 — HiMIC-Monthly: A 1 km high-resolution atmospheric moisture index collection over China, 2003–2020 [file 41597_2024_3230_MOESM1_ESM.pdf]

Supplementary Information

A 1 km high-resolution atmospheric moisture index collection over China, 2003–2020

Hui Zhang[1], Ming Luo[1,2*], Wenfeng Zhan[3], Yongquan Zhao[4], Yuanjian Yang[5], Erjia Ge[6], Guicai Ning[5], Jing Cong[7]

1. Guangdong Provincial Key Laboratory of Urbanization and Geo-simulation, School of Geography and Planning, Sun Yat-sen University, Guangzhou 51006, China.
2. Institute of Environment, Energy and Sustainability, The Chinese University of Hong Kong, Shatin, Hong Kong SAR, China.
3. Jiangsu Provincial Key Laboratory of Geographic Information Science and Technology, International Institute for Earth System Science, Nanjing University, Nanjing 210023, China.
4. Key Laboratory of Watershed Geographic Sciences, Nanjing Institute of Geography and Limnology, Chinese Academy of Sciences, Nanjing 210008, China.
5. School of Atmospheric Physics, Nanjing University of Information Science & Technology, Nanjing 210044, China.
6. Dalla Lana School of Public Health, University of Toronto, Toronto, Ontario M5T 3M7, Canada.
7. Tianjin Municipal Meteorological Observatory, Tianjin 300074, China.

*Correspondence: Dr. Ming Luo, School of Geography and Planning, Sun Yat-sen University, Guangzhou 510006, China, email: luom38@mail.sysu.edu.cn

Figures

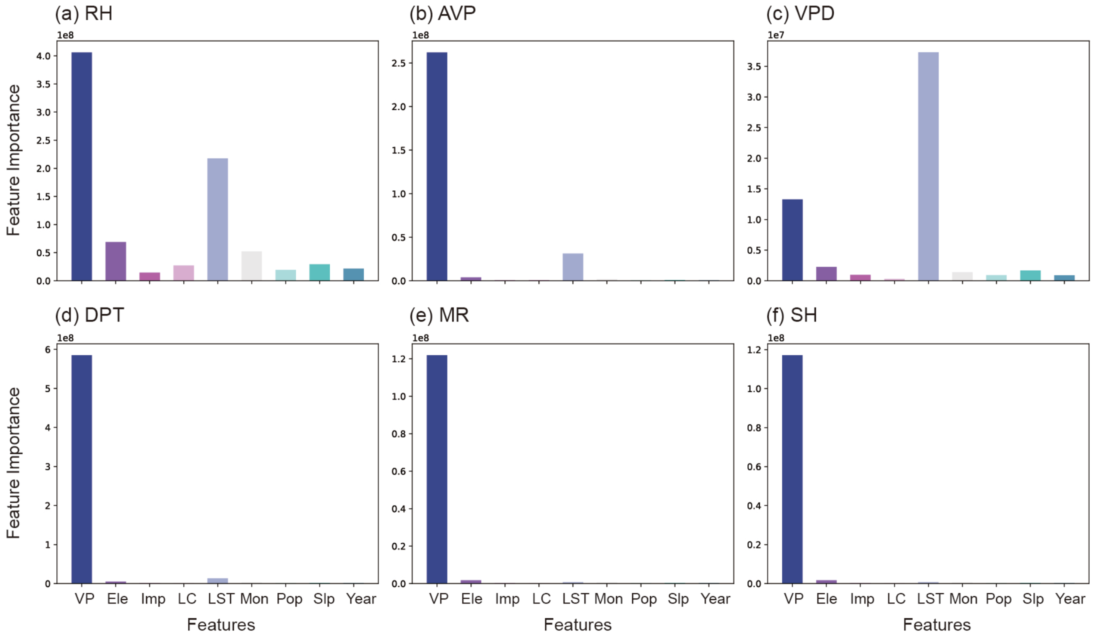

Figure S1. The importance of nine covariates in predicting six moisture indices: (a) RH, (b) AVP, (c) VPD, (d) DPT, (e) MR, and (f) SH. VP, Ele, Imp, LC, LST, Mon, Pop, Slp, and Year represent vapor pressure, elevation, impervious surface, land cover, land surface temperature, month of the year, population density, slope, and year, respectively.

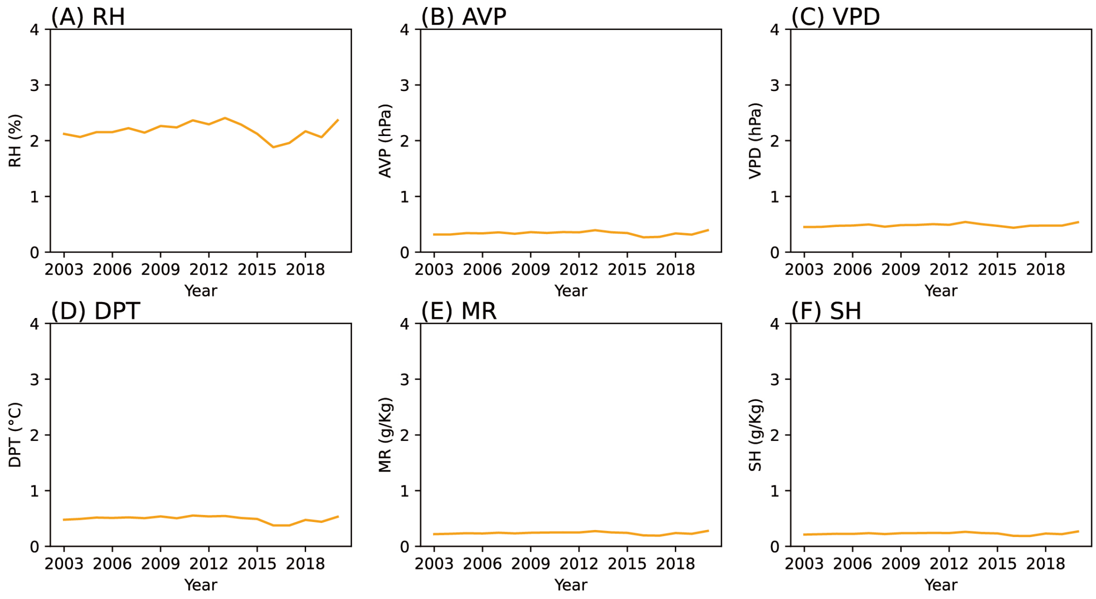

Figure S2. Annual mean absolute error (MAE) values of the six moisture indices over the mainland of China from 2003 to 2020.

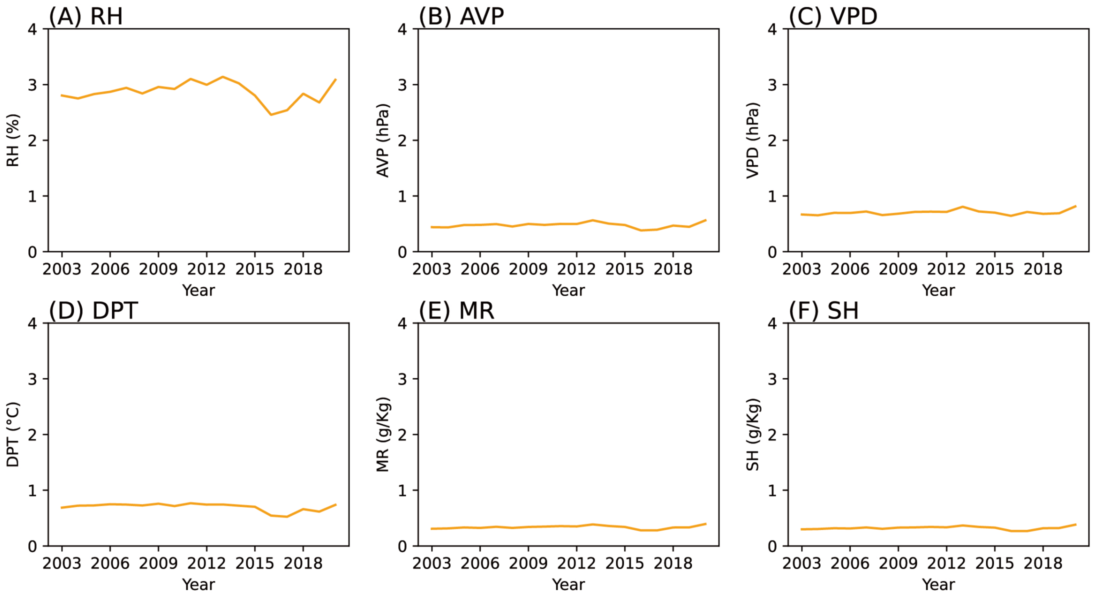

Figure S3. As Figure S2 but for root mean square error (RMSE).

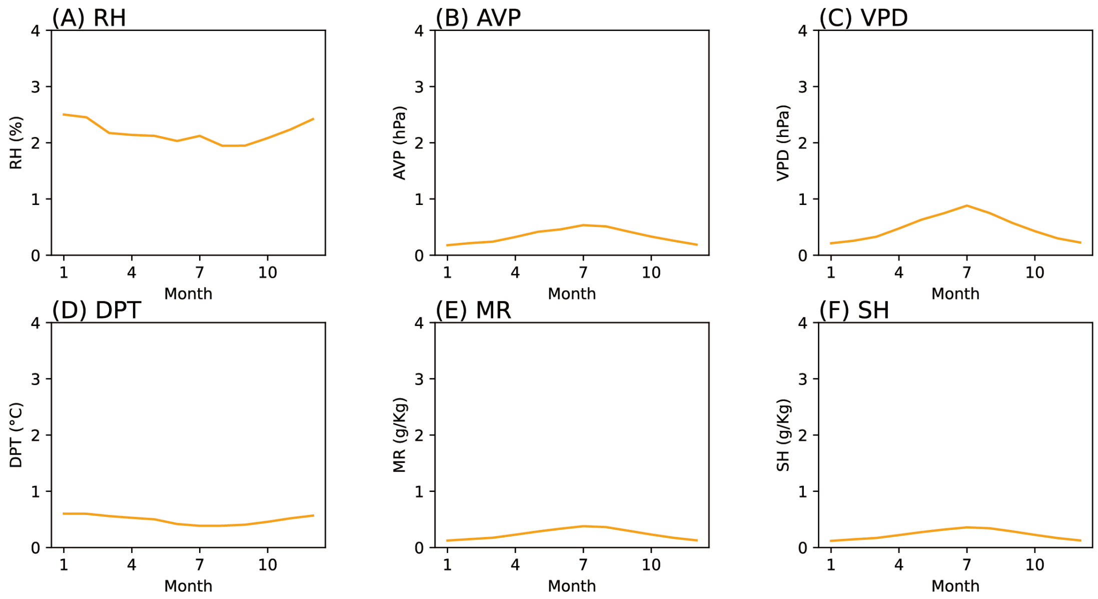

Figure S4. Monthly MAE values of the six moisture indices over the mainland of China from 2003 to 2020.

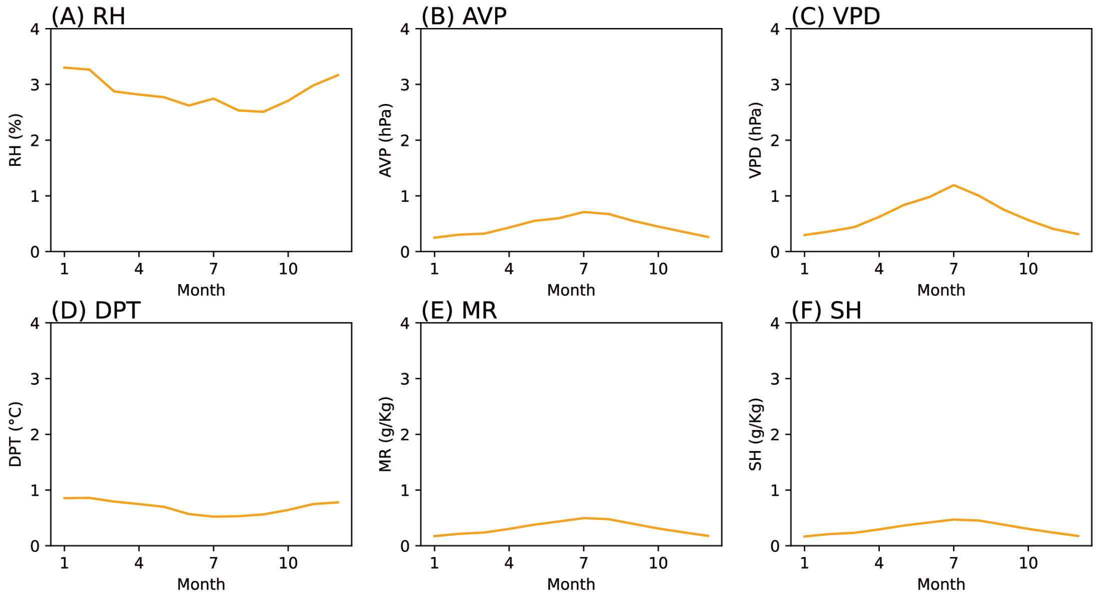

Figure S5. As Figure S4 but for RMSE.

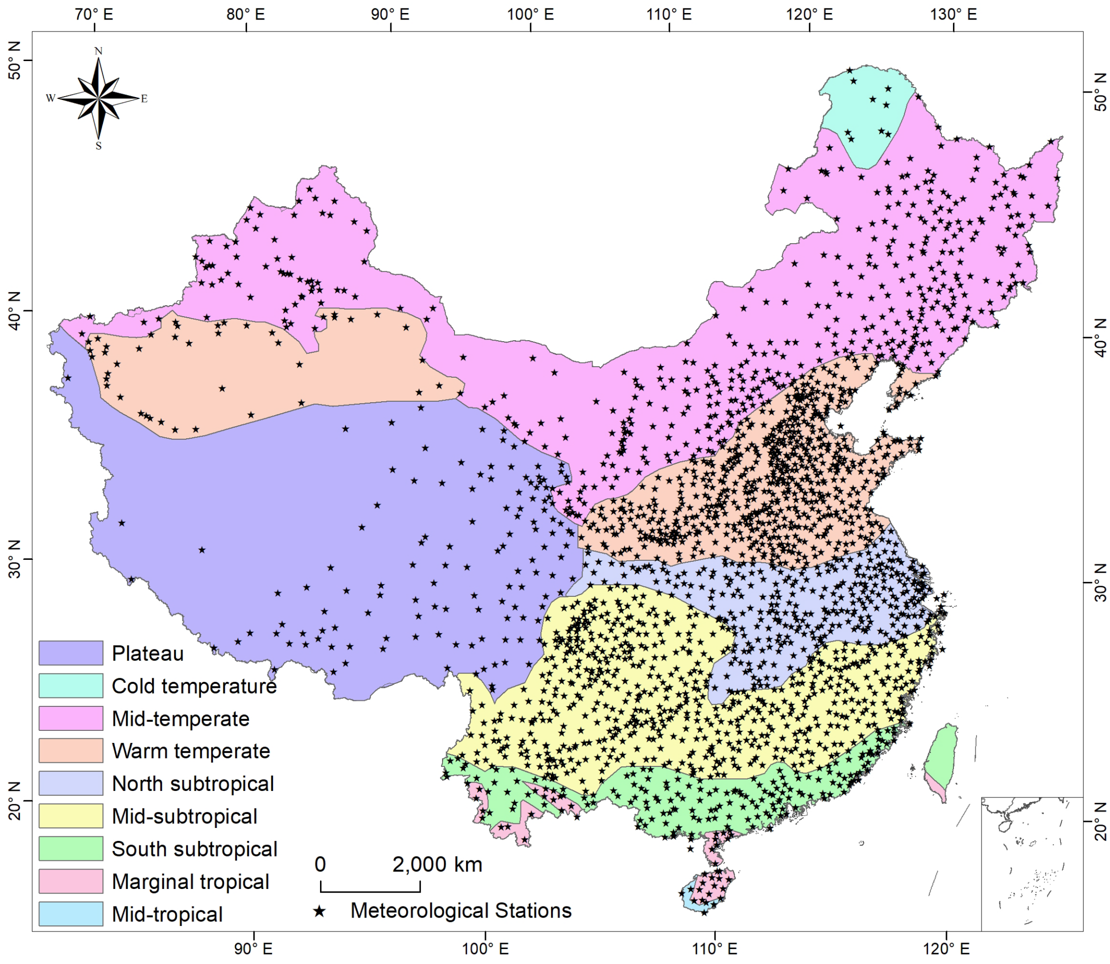

Figure S6. Climate zones over the mainland of China.

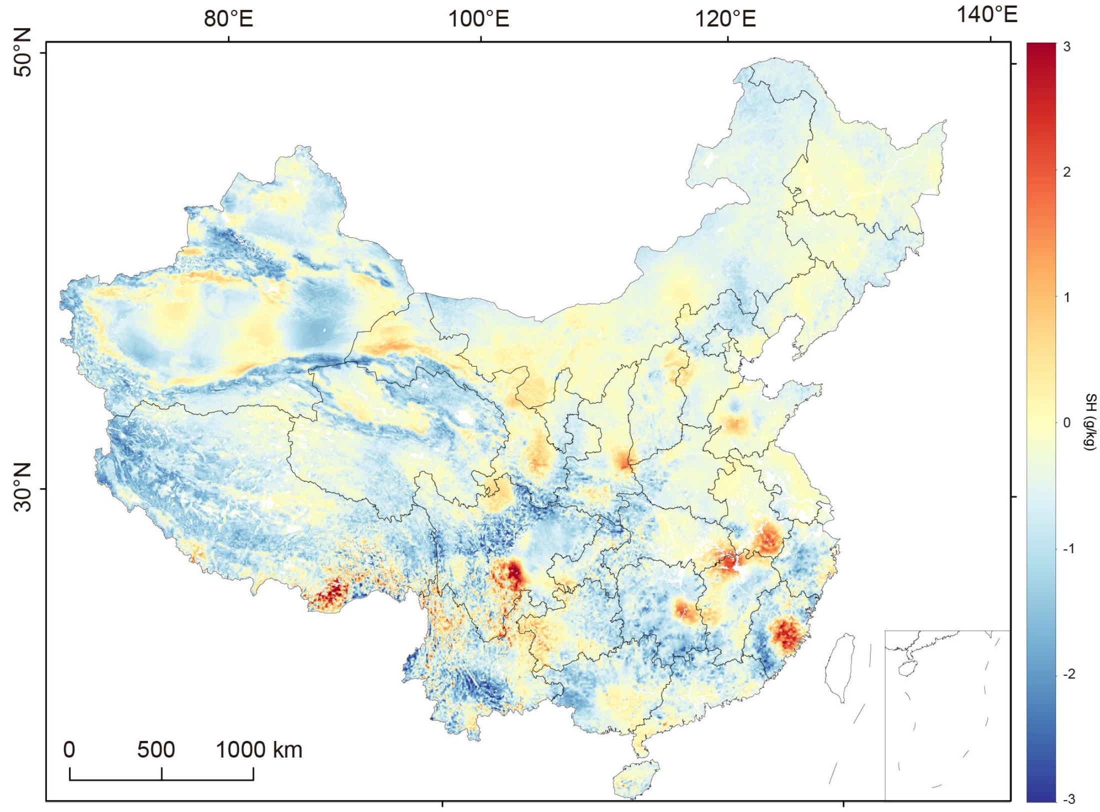

Figure S7. The difference in SH between CMFD and HiMIC-Monthly datasets (i.e., CMFD minus HiMIC-Monthly) from 2003 to 2018 over the mainland of China.

Tables

Table S1. Overall accuracies of the six moisture indices from 2003 to 2020 predicted by the inclusion of wind speed as a covariate.

**Moisture Indices**	**R^2**	**MAE**	**RMSE**
RH (%)	0.959	2.200	2.890
AVP (hPa)	0.997	0.349	0.493
VPD (hPa)	0.965	0.486	0.714
DPT (°C)	0.996	0.502	0.717
MR (g/kg)	0.996	0.241	0.341
SH (g/kg)	0.996	0.233	0.329

Table S2. Overall accuracies of the six moisture indices from 2003 to 2020 predicted by different machine learning methods.

**Moisture Indices**	**Models**	**R^2**	**MAE**	**RMSE**
RH (%)	LightGBM	0.960	2.182	2.870
	XGBoost	0.955	2.317	3.033
	CatBoost	0.943	2.636	3.422
	RandomForest	0.767	5.242	6.908
AVP (hPa)	LightGBM	0.997	0.338	0.478
	XGBoost	0.996	0.363	0.509
	CatBoost	0.995	0.420	0.587
	RandomForest	0.982	0.801	1.122
VPD (hPa)	LightGBM	0.965	0.483	0.707
	XGBoost	0.960	0.520	0.751
	CatBoost	0.951	0.583	0.832
	RandomForest	0.823	1.10	1.588
DPT (°C)	LightGBM	0.997	0.495	0.703
	XGBoost	0.996	0.521	0.736
	CatBoost	0.995	0.597	0.820
	RandomForest	0.978	1.198	1.77
MR (g/kg)	LightGBM	0.996	0.238	0.337
	XGBoost	0.993	0.330	0.458
	CatBoost	0.995	0.290	0.406
	RandomForest	0.980	0.567	0.796
SH (g/kg)	LightGBM	0.996	0.229	0.324
	XGBoost	0.993	0.324	0.448
	CatBoost	0.995	0.284	0.396
	RandomForest	0.979	0.556	0.778

Table S3. Overall accuracies of the six moisture indices from 2003 to 2020 predicted by using the left-out-station method.

**Moisture Indices**	**R^2**	**MAE**	**RMSE**
RH (%)	0.862	4.031	5.335
AVP (hPa)	0.988	0.664	0.944
VPD (hPa)	0.877	0.904	1.332
DPT (°C)	0.987	0.943	1.357
MR (g/kg)	0.985	0.461	0.658
SH (g/kg)	0.986	0.448	0.642

Table S4. Annual mean *MAE* between the observed and predicted moisture values over the mainland of China from 2003 to 2020.

Year	RH (%)	AVP (hPa)	VPD (hPa)	DPT (°C)	MR (g/kg)	SH (g/kg)
2003	2.122	0.317	0.452	0.478	0.219	0.212
2004	2.066	0.317	0.452	0.493	0.226	0.219
2005	2.152	0.342	0.472	0.517	0.236	0.226
2006	2.154	0.337	0.478	0.510	0.231	0.224
2007	2.225	0.355	0.497	0.520	0.245	0.237
2008	2.144	0.329	0.457	0.506	0.233	0.221
2009	2.264	0.359	0.485	0.538	0.245	0.236
2010	2.238	0.344	0.488	0.504	0.248	0.238
2011	2.365	0.360	0.502	0.554	0.251	0.242
2012	2.293	0.355	0.490	0.538	0.250	0.239
2013	2.408	0.394	0.542	0.545	0.274	0.261
2014	2.289	0.357	0.501	0.509	0.251	0.240
2015	2.124	0.343	0.472	0.492	0.242	0.233
2016	1.883	0.266	0.438	0.376	0.197	0.189
2017	1.959	0.274	0.473	0.376	0.193	0.186
2018	2.169	0.336	0.477	0.475	0.239	0.231
2019	2.062	0.315	0.476	0.441	0.225	0.219
2020	2.366	0.393	0.537	0.532	0.278	0.268

Table S5. Annual mean *RMSE* between the observed and predicted moisture values over the mainland of China from 2003 to 2020.

**Year**	**RH (%)**	**AVP (hPa)**	**VPD (hPa)**	**DPT (°C)**	**MR (g/kg)**	**SH (g/kg)**
2003	2.803	0.441	0.668	0.688	0.309	0.299
2004	2.750	0.436	0.654	0.723	0.315	0.304
2005	2.829	0.479	0.698	0.727	0.332	0.318
2006	2.870	0.481	0.695	0.749	0.324	0.314
2007	2.941	0.496	0.722	0.743	0.344	0.332
2008	2.840	0.453	0.658	0.727	0.324	0.308
2009	2.957	0.499	0.683	0.758	0.341	0.330
2010	2.921	0.481	0.714	0.716	0.348	0.333
2011	3.101	0.499	0.719	0.767	0.356	0.342
2012	2.996	0.497	0.715	0.742	0.351	0.335
2013	3.139	0.564	0.807	0.744	0.387	0.368
2014	3.022	0.504	0.722	0.723	0.359	0.342
2015	2.803	0.480	0.701	0.703	0.341	0.329
2016	2.457	0.382	0.644	0.545	0.280	0.269
2017	2.540	0.397	0.714	0.524	0.280	0.267
2018	2.836	0.469	0.680	0.661	0.332	0.318
2019	2.679	0.447	0.690	0.616	0.333	0.321
2020	3.085	0.563	0.813	0.738	0.394	0.382

Table S6. Monthly mean *MAE* between the observed and predicted moisture values over the mainland of China from 2003 to 2020.

**Month**	**RH (%)**	**AVP (hPa)**	**VPD (hPa)**	**DPT (°C)**	**MR (g/kg)**	**SH (g/kg)**
Jan	2.502	0.178	0.212	0.602	0.123	0.118
Feb	2.452	0.215	0.257	0.600	0.149	0.145
Mar	2.173	0.240	0.328	0.558	0.173	0.169
Apr	2.140	0.324	0.475	0.528	0.228	0.221
May	2.123	0.416	0.633	0.501	0.285	0.274
Jun	2.032	0.460	0.748	0.417	0.335	0.320
Jul	2.122	0.534	0.882	0.386	0.379	0.358
Aug	1.946	0.512	0.750	0.387	0.363	0.342
Sep	1.949	0.419	0.575	0.406	0.297	0.286
Oct	2.083	0.331	0.427	0.457	0.231	0.225
Nov	2.235	0.257	0.300	0.519	0.171	0.167
Dec	2.421	0.188	0.225	0.567	0.127	0.125

Table S7. Monthly mean *RMSE* between the observed and predicted moisture values over the mainland of China from 2003 to 2020.

**Month**	**RH (%)**	**AVP (hPa)**	**VPD (hPa)**	**DPT (°C)**	**MR (g/kg)**	**SH (g/kg)**
Jan	3.300	0.249	0.296	0.855	0.173	0.166
Feb	3.266	0.304	0.361	0.859	0.215	0.210
Mar	2.874	0.322	0.439	0.792	0.237	0.232
Apr	2.818	0.430	0.622	0.748	0.302	0.293
May	2.771	0.550	0.837	0.698	0.377	0.363
Jun	2.620	0.599	0.976	0.569	0.436	0.417
Jul	2.745	0.709	1.193	0.522	0.496	0.470
Aug	2.533	0.674	1.004	0.530	0.477	0.453
Sep	2.509	0.549	0.753	0.562	0.392	0.378
Oct	2.708	0.447	0.564	0.642	0.309	0.302
Nov	2.980	0.354	0.406	0.748	0.241	0.236
Dec	3.166	0.262	0.313	0.779	0.177	0.175

Table S8. *MAE* of the six predicted moisture indices across different climate zones of China.

**Climate zones**	**RH (%)**	**AVP (hPa)**	**VPD (hPa)**	**DPT (°C)**	**MR (g/kg)**	**SH (g/kg)**
Cold temperature	2.312	0.249	0.283	0.690	0.140	0.134
Mid-temperate	2.435	0.282	0.405	0.639	0.198	0.193
Warm temperate	2.128	0.315	0.459	0.499	0.217	0.209
North subtropical	1.958	0.354	0.506	0.370	0.237	0.225
Mid-subtropical	2.090	0.382	0.548	0.395	0.270	0.258
South subtropical	1.895	0.408	0.579	0.352	0.282	0.266
Marginal tropical	1.898	0.449	0.603	0.366	0.316	0.302
Mid-tropical	1.963	0.507	0.698	0.347	0.332	0.320
Plateau	3.000	0.291	0.361	0.952	0.261	0.259

Table S9. *RMSE* of the six predicted moisture indices across different climate zones.

**Climate zones**	**RH (%)**	**AVP (hPa)**	**VPD (hPa)**	**DPT (°C)**	**MR (g/kg)**	**SH (g/kg)**
Cold temperature	2.926	0.386	0.448	0.913	0.222	0.215
Mid-temperate	3.162	0.416	0.623	0.860	0.291	0.285
Warm temperate	2.812	0.453	0.689	0.692	0.312	0.299
North subtropical	2.569	0.507	0.742	0.502	0.344	0.326
Mid-subtropical	2.708	0.521	0.769	0.529	0.370	0.353
South subtropical	2.462	0.533	0.781	0.469	0.370	0.350
Marginal tropical	2.494	0.592	0.832	0.493	0.421	0.404
Mid-tropical	2.745	0.644	1.031	0.457	0.427	0.413
Plateau	3.915	0.408	0.509	1.316	0.364	0.364

Table S10. R^2 of the six predicted moisture indices across different climate zones of China.

**Climate zones**	**RH (%)**	**AVP (hPa)**	**VPD (hPa)**	**DPT (°C)**	**MR (g/kg)**	**SH (g/kg)**
Cold temperature	0.922	0.996	0.964	0.997	0.997	0.997
Mid-temperate	0.950	0.995	0.982	0.995	0.995	0.995
Warm temperate	0.955	0.997	0.974	0.996	0.996	0.996
North subtropical	0.888	0.996	0.939	0.997	0.996	0.996
Mid-subtropical	0.906	0.995	0.941	0.995	0.994	0.994
South subtropical	0.896	0.995	0.905	0.994	0.994	0.994
Marginal tropical	0.909	0.992	0.907	0.991	0.989	0.990
Mid-tropical	0.845	0.984	0.840	0.984	0.984	0.984
Plateau	0.939	0.988	0.963	0.984	0.988	0.988

Table S11. R^2 of the six predicted moisture indices in the 20 major urban agglomerations (UAs) of the mainland of China from 2003 to 2020.

**UAs**	**RH (%)**	**AVP (hPa)**	**VPD (hPa)**	**DPT (°C)**	**MR (g/kg)**	**SH (g/kg)**
Beibu Gulf	0.853	0.995	0.889	0.994	0.994	0.995
Beijing-Tianjin-Hebei	0.959	0.997	0.973	0.997	0.997	0.997
Central Guizhou	0.794	0.995	0.917	0.996	0.995	0.995
Central Henan	0.946	0.997	0.966	0.997	0.996	0.997
Central Shanxi	0.954	0.996	0.975	0.995	0.996	0.996
Central Yunnan	0.949	0.992	0.958	0.990	0.992	0.993
Chengdu-Chongqing	0.825	0.993	0.926	0.993	0.992	0.993
Guanzhong	0.928	0.995	0.962	0.996	0.994	0.995
Harbin-Changchun	0.942	0.998	0.974	0.998	0.997	0.998
Hu-Bao-E-Yu	0.932	0.994	0.984	0.995	0.994	0.994
Jiang-Huai	0.896	0.997	0.941	0.997	0.997	0.997
Lanzhou-Xining	0.921	0.993	0.965	0.993	0.993	0.993
Mid-southern Liaoning	0.932	0.996	0.954	0.997	0.996	0.997
Middle Reaches of Yangtze River	0.859	0.996	0.940	0.997	0.996	0.996
Ningxia Yellow River	0.949	0.996	0.984	0.995	0.995	0.995
North Tianshan Mountain	0.964	0.992	0.991	0.993	0.992	0.992
Pearl River Delta	0.919	0.996	0.902	0.996	0.995	0.996
Shandong Peninsula	0.948	0.997	0.965	0.997	0.997	0.997
West Coast of Taiwan Strait	0.872	0.994	0.924	0.994	0.994	0.994
Yangtze River Delta	0.894	0.997	0.950	0.998	0.997	0.998

Table S12. *MAE* of the six predicted moisture indices in the 20 major urban agglomerations (UAs) of the mainland of China from 2003 to 2020.

UAs	RH (%)	AVP (hPa)	VPD (hPa)	DPT (°C)	MR (g/Kg)	SH (g/Kg)
Beibu Gulf	1.893	0.416	0.600	0.341	0.287	0.266
Beijing-Tianjin-Hebei	2.048	0.298	0.431	0.505	0.198	0.191
Central Guizhou	2.064	0.318	0.465	0.345	0.230	0.220
Central Henan	2.056	0.330	0.495	0.437	0.214	0.205
Central Shanxi	2.199	0.300	0.423	0.582	0.207	0.202
Central Yunnan	2.028	0.311	0.483	0.410	0.246	0.224
Chengdu-Chongqing	2.218	0.425	0.585	0.421	0.294	0.281
Guanzhong	2.388	0.348	0.487	0.502	0.255	0.242
Harbin-Changchun	2.068	0.233	0.326	0.468	0.158	0.151
Hu-Bao-E-Yu	2.421	0.254	0.421	0.643	0.193	0.192
Jiang-Huai	1.874	0.344	0.476	0.350	0.220	0.214
Lanzhou-Xining	2.627	0.270	0.395	0.604	0.222	0.219
Mid-southern Liaoning	2.394	0.322	0.427	0.534	0.215	0.204
Middle Reaches of Yangtze River	1.978	0.375	0.559	0.361	0.253	0.242
Ningxia Yellow River	2.098	0.252	0.411	0.563	0.207	0.204
North Tianshan Mountain	2.315	0.292	0.456	0.653	0.193	0.185
Pearl River Delta	1.589	0.374	0.561	0.296	0.257	0.245
Shandong Peninsula	1.901	0.307	0.449	0.418	0.207	0.199
West Coast of Taiwan Strait	1.959	0.441	0.588	0.395	0.291	0.277
Yangtze River Delta	1.645	0.308	0.459	0.298	0.196	0.182

Table S13. *RMSE* of the six predicted moisture indices in the 20 major urban agglomerations (UAs) of the mainland of China from 2003 to 2020.

**UAs**	**RH (%)**	**AVP (hPa)**	**VPD (hPa)**	**DPT (°C)**	**MR (g/Kg)**	**SH (g/Kg)**
Beibu Gulf	2.503	0.540	0.794	0.452	0.371	0.344
Beijing-Tianjin-Hebei	2.655	0.431	0.632	0.687	0.288	0.278
Central Guizhou	2.622	0.423	0.641	0.445	0.313	0.299
Central Henan	2.645	0.467	0.710	0.585	0.313	0.298
Central Shanxi	2.827	0.432	0.615	0.811	0.292	0.282
Central Yunnan	2.709	0.408	0.653	0.537	0.328	0.302
Chengdu-Chongqing	2.892	0.590	0.837	0.560	0.414	0.392
Guanzhong	3.057	0.486	0.698	0.658	0.359	0.341
Harbin-Changchun	2.704	0.354	0.508	0.609	0.245	0.234
Hu-Bao-E-Yu	3.121	0.369	0.631	0.824	0.277	0.274
Jiang-Huai	2.413	0.485	0.695	0.461	0.321	0.308
Lanzhou-Xining	3.335	0.374	0.559	0.830	0.301	0.294
Mid-southern Liaoning	3.099	0.475	0.625	0.691	0.306	0.293
Middle Reaches of Yangtze River	2.575	0.534	0.807	0.479	0.365	0.348
Ningxia Yellow River	2.704	0.365	0.621	0.752	0.302	0.296
North Tianshan Mountain	3.014	0.398	0.693	0.859	0.261	0.250
Pearl River Delta	2.083	0.500	0.791	0.392	0.334	0.316
Shandong Peninsula	2.496	0.441	0.659	0.551	0.307	0.294
West Coast of Taiwan Strait	2.493	0.585	0.805	0.521	0.387	0.368
Yangtze River Delta	2.116	0.451	0.699	0.391	0.290	0.270
